# Declining lung cancer mortality of young Australian women despite increased smoking is linked to reduced cigarette ‘tar’ yields

**DOI:** 10.1054/bjoc.2000.1558

**Published:** 2001-02

**Authors:** L Blizzard, T Dwyer

**Affiliations:** Menzies Centre for Population Health Research, University of Tasmania, GPO Box 252–23, Hobart, 7001, Australia

**Keywords:** lung cancer, mortality, women, epidemiology, tobacco

## Abstract

Lung cancer data were examined to determine whether the mortality rates of young Australian women have continued to increase in line with the proportions of them who have smoked tobacco. Trends in annual age-specific lung cancer mortality were estimated for 1965–1998. Age-specific mortality rates and age-adjusted ratios of mortality rates were calculated for birth cohorts. Proportions of smokers in those cohorts were estimated from results of eight national surveys of smoking, and their mean ages of commencement and years of smoking were assessed from surveys of smokers in two states. Lung cancer mortality rates of 20–44-year-old Australian women peaked in 1986. Age-adjusted mortality rates are lower for women born in the 1950s and 1960s than for women born in the 1940s, despite higher proportions of smokers, younger age of commencement and longer duration of smoking by age 30 years in the more recent cohorts. Increased smoking has not resulted in higher lung cancer mortality for Australian women born in the 1950s and 1960s. Reductions in tar yields of Australian-made cigarettes, which would have affected primarily those born after the 1940s, may be responsible. © 2001 Cancer Research Campaign http://www.bjcancer.com


*British Journal of Cancer* (2001) **84**(3), 392–396
© 2001 Cancer Research Campaign
doi: 10.1054/ bjoc.2000.1558, available online at http://www.idealibrary.com on IDEAL®

http://www.bjcancer.com

### Declining lung cancer mortality of young Australian women despite increased smoking is linked to reduced cigarette 'tar' yields

**L Blizzard and T Dwyer**

Menzies Centre for Population Health Research, University of Tasmania, GPO Box 252–23, Hobart 7001, Australia

**Summary** Lung cancer data were examined to determine whether the mortality rates of young Australian women have continued to increase in line with the proportions of them who have smoked tobacco. Trends in annual age-specific lung cancer mortality were estimated for 1965–1998. Age-specific mortality rates and age-adjusted ratios of mortality rates were calculated for birth cohorts. Proportions of smokers in those cohorts were estimated from results of eight national surveys of smoking, and their mean ages of commencement and years of smoking were assessed from surveys of smokers in two states. Lung cancer mortality rates of 20–44-year-old Australian women peaked in 1986. Age-adjusted mortality rates are lower for women born in the 1950s and 1960s than for women born in the 1940s, despite higher proportions of smokers, younger age of commencement and longer duration of smoking by age 30 years in the more recent cohorts. Increased smoking has not resulted in higher lung cancer mortality for Australian women born in the 1950s and 1960s. Reductions in tar yields of Australian-made cigarettes, which would have affected primarily those born after the 1940s, may be responsible.
© 2001 Cancer Research Campaign http://www.bjcancer.com

**Keywords**: lung cancer; mortality; women; epidemiology; tobacco

In England and Wales (Doll, 1989) and the USA (Doll, 1991) lung cancer incidence for 20–44-year-old women ceased rising in the 1970s, before reaching the higher rates for their male counterparts. The subsequent decline in deaths of young women from lung cancer in those countries has been associated with declining prevalence of tobacco smoking in the birth cohorts with reduced mortality, and with reductions in the 'tar' yields of the cigarettes they smoked (Doll, 1991).

The objective of this study was to determine whether similar lung cancer mortality trends could be identified in Australia, where increasing numbers of women continued to take up smoking prior to the 1990s (Hill et al, 1998). Trends in the rates of 20–44-year-old Australians are examined to assess future prospects (Doll, 1991). Analyses for older age groups are presented for comparison, and because declines in lung cancer rates have extended to women aged in their 50s in England and Wales (Doll, 1991; Gilliland and Samet, 1994), and to 40–49-year-old women in the USA (Wingo et al, 1999).

To enable inferences to be drawn about the contribution of trends in tobacco smoking to mortality, estimates are made of the proportions of smokers in the birth cohorts at risk. Information on age of commencement and duration of smoking was also sought because risk of lung cancer for smoking depends principally on duration of smoking (Doll and Peto, 1978), and early age of commencement may independently increase risk (Hegmann et al, 1993).

*Received 8 June 2000*
*Revised 20 September 2000*
*Accepted 3 October 2000*

*Correspondence to:* L Blizzard. E-mail: Leigh.Blizzard@utas.edu.au

### METHODS

### Lung cancer mortality rates, 1965–1998

Annual data on deaths from carcinoma of the trachea, bronchus and lung (lung cancer, ICD 162) in Australia during 1965–1998 were provided in gender and 5-year age groups by the Australian Bureau of Statistics. Population data were obtained from the same source.

Annual age-specific mortality rates were calculated. For visual clarity in Figures 1 and 2, mortality and population data were pooled for adjacent 2-year intervals. Trends in annual rates were estimated by linear regression of the age-specific rates on year of diagnosis. The probabilities values reported refer to the results of including a term for the square of year as a test of curvature.

### Trends in mortality rates by birth cohort

Age-specific rates of lung cancer mortality were calculated for 5-year birth cohorts. Because there were few deaths among persons younger than 25 years, the analyses were restricted to those aged ≥ 25 years at time of death.

Age-adjusted rates were estimated by modelling the mortality data in categories of age, year of death (period) and year of birth (cohort) under standard assumptions (log-linear relationship, Poisson errors, logarithm of population entered as an offset, maximum likelihood fitting). Because the data for 85+-year-olds were provided in a single grouping, these analyses were restricted to those aged 25–84 years at time of death. Annual observations were grouped into 4-year (1965–1968) or 5-year (1969–1973, 1974–1978, 1979–1983, 1984–1988, 1989–1993, 1994–1998) intervals, and birth cohort was calculated as cohort = period

(mid-point) − age (mid-point). Each birth cohort is referred to by its mid-point. The estimates obtained were ratios of their age-adjusted lung cancer mortality rate relative to that of a reference cohort. Hierarchical model fitting strategies were used to test the fit of models (Clayton and Schifflers, 1987). Each of the three factors (age, period, cohort) contributed significantly ($P < 0.001$) to model fit for 1965–1998 but the estimates were not unique because of the exact linear dependence (cohort = period − age) between them (Clayton and Schifflers, 1987). The approach used was to estimate age-cohort models for sub-intervals of time in which period changes did not contribute to temporal trends. The intervals used, and the results of tests of the improvement in model fit from adding predictors for period, were 1965–1983 (men $P = 0.090$, women $P = 0.400$) and 1979–1998 (men $P = 0.640$, women $P = 0.156$). For women, there was no evidence of an (age–cohort) interaction in either the earlier ($P = 0.569$) or later ($P = 0.170$) epochs. After weighting by the number of deaths, the two stratum-specific mortality rate estimates for cohorts were combined by averaging.

### Prevalence of smoking

Estimates of percentages of ‘ever-smokers’ (current and former smokers) and former smokers were calculated from published results of eight national surveys undertaken with a standard method in 1974, 1976, 1980, 1983, 1986, 1989, 1992 and 1995 (Hill et al, 1998). To obtain estimates for 5-year birth cohorts, we required prevalence estimates for the relevant 5-year age-groups at 5-yearly intervals. We chose 1980 and 1995 as two of the years and obtained estimates for 1975, 1985 and 1990 by pooling data for the two closest years to each after weighting for proximity to the year of interest. The youngest age-group included was 20–24-year-olds, and the oldest was 60–64-year-olds for whom the survey estimates for 60–69-year-olds were used. Analyses were restricted to birth cohorts with at least three prevalence estimates. Percentages of ever-smokers were calculated by pooling estimates for each cohort. Percentages of former smokers were calculated from the positive relationship between former smoker proportions and increasing age that was estimated by linear regression for each cohort. Percentages of current smokers aged 30–34 years were calculated by subtracting the regression estimate of the percentage of former smokers at age 30–34 years from the percentage of ever-smokers. Choosing this age-group allowed the percentage of current smokers at a common age to be compared for post-1930s birth cohorts.

### Age of commencement of smoking

Representative Australian data on age of commencement of smoking are limited. This information is not routinely collected in the national surveys, but was for the 583 ever-smokers in the New South Wales sample ($n = 1998$) of the 1995 national survey. The Anti-Cancer Council of Victoria provided summary data in 5-year age-groups, together with results for 556 ever-smokers in a separate survey ($n = 2331$) of smoking in Victoria in 1987. These two states account for nearly 60% of the population of Australia.

Mean age of commencement was calculated for each 5-year birth cohort. For the samples combined, mean years of smoking to age 30 years were estimated from the distributions of values of years of smoking and either current age (current smokers) or age at quitting (former smokers). Analyses were restricted to cohorts that had reached 30 years-of-age. The relationships between birth year and mean age of commencement or mean years of smoking to age 30 were estimated by linear regression.

### RESULTS

### Lung cancer mortality rates, 1965–1998

The trends in biennial age-specific lung cancer mortality rates for 45+-year-old Australians during 1965–1998 are depicted in Figure 1. Rates for 45+-year-old men have declined since the early 1980s ($P < 0.001$). Rates continued to increase for 45+-year-old women, but at a decreasing pace ($P < 0.001$) for 45–54, 55–64 and 65–74-year-olds.

Figure 2 depicts biennial rates of lung cancer mortality for 20–44-year-olds. Rates for young men declined throughout the period and at an increasing pace ($P = 0.027$), but those for young women peaked in 1986 and have declined since ($P = 0.017$).

### Trends in mortality rates by birth cohort

A birth cohort analysis was undertaken to identify the birth cohorts of women with reduced lung cancer mortality rates. Age-specific rates are shown for each birth cohort in Figure 3 (Panels A and B). Ratios of age-adjusted rates of birth cohorts relative to a reference cohort are shown in Panels C and D. Together, these results suggest that age-adjusted mortality rates have declined in post-1910 birth cohorts of men and in post-1940s birth cohorts of women.

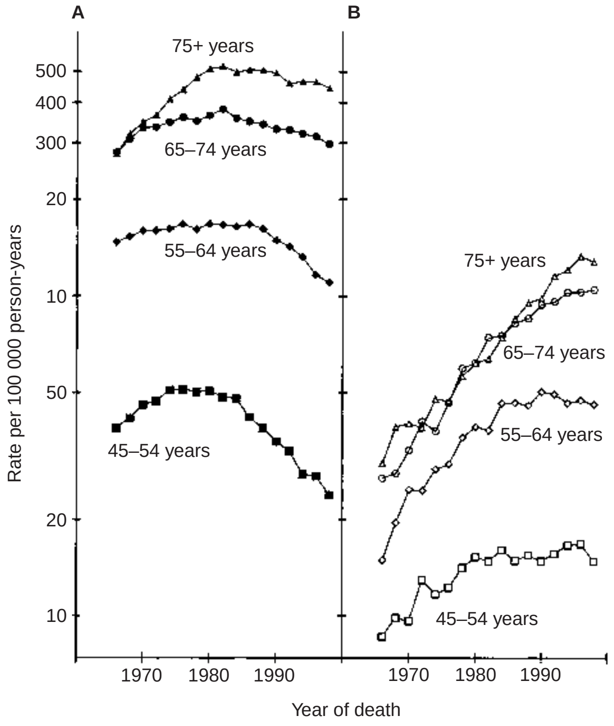

**Figure 1** Age-specific rates of lung cancer mortality for 45+ year old (**A**) men and (**B**) women, Australia 1965–1998

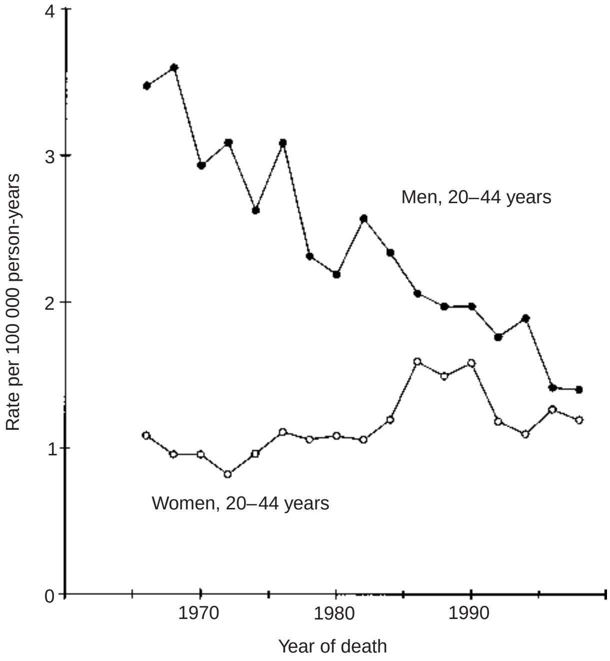

**Figure 2** Rates of lung cancer mortality for 20–44 year olds, Australia 1965–98

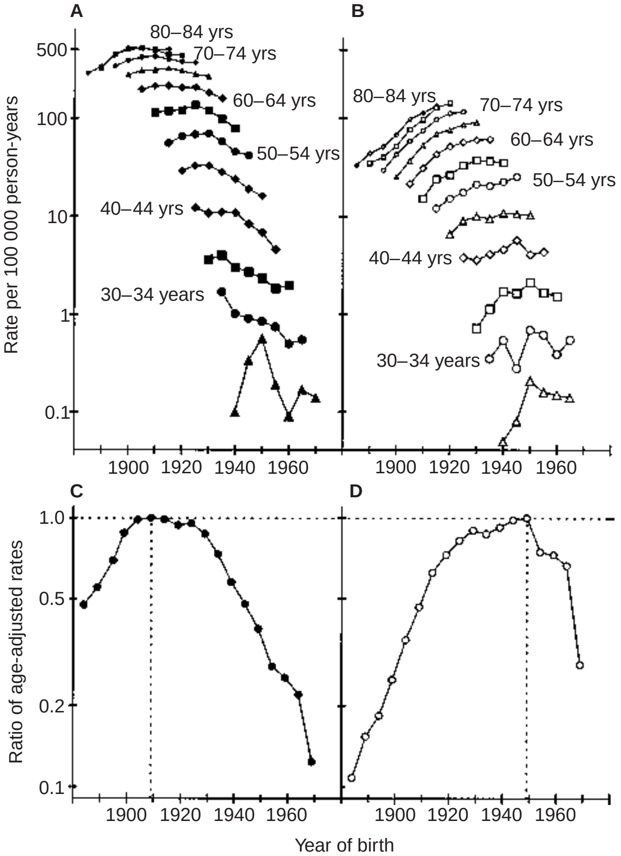

**Figure 3** Lung cancer mortality by year of birth, Australia 1965–1998: age-specific rates for men (**A**) and women (**B**), and age-adjusted rates for men (**C**) and women (**D**) relative to the birth cohorts with the highest rates.

### Prevalence of smoking

Estimates of smoking prevalence in 5-year birth cohorts of Australians are shown in Figure 4. In most successive birth cohorts, the percentages of ever-smokers (Panel A) declined for men but increased for women, reaching parity among those born in the 1960s. There has been some increase in the percentage of women who have quit smoking (Panel B). Nevertheless, when

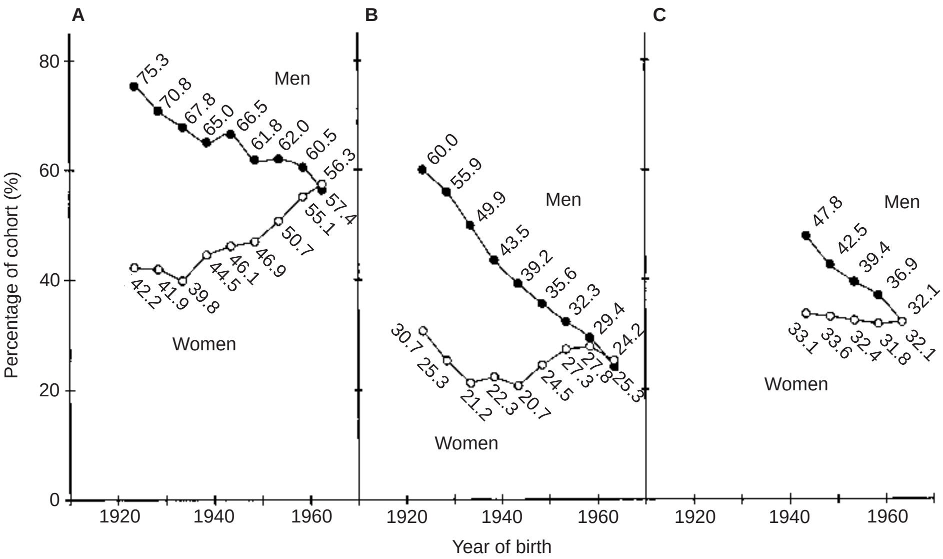

**Figure 4** Prevalence of smoking by year of birth, Australia 1995: percentages of ever-smokers (**A**), former smokers (**B**) and current smokers when aged 30–34 years (**C**)

*© 2001 Cancer Research Campaign*

percentages of current smokers aged 30–34 years are calculated as a common measure of smoking prevalence, no decrease in smoking can be seen (Panel C) in the birth cohorts of women with lower lung cancer mortality.

### Age of commencement of smoking

The estimates of mean age of commencement of smoking in Figure 5 (Panel A) show a trend to younger age of uptake in more recent birth cohorts (men $P < 0.01$, women $P < 0.01$) of smokers in the samples from the Australian states of Victoria and New South Wales. Also shown in Figure 5 are estimates of their mean years of smoking (Panel B). When years of smoking by age 30 were calculated (data not shown) as a measure of common duration for subjects aged 30–69 years, there was a trend of increasing years of smoking for more recently-born women ($P < 0.01$).

### DISCUSSION

Lung cancer mortality rates for 20–44-year-old Australian women peaked in 1986, before reaching the level for male rates. The subsequent fall in their mortality is consistent with the declining rates for 20–44-year-old women in England and Wales (Doll, 1989; Gilliland and Samet, 1994) and the USA (Doll, 1991; Wingo et al, 1999), but delayed by about a decade. Rates for the young Australian man are falling rapidly, and the observation (Dwyer et al, 1994) of higher female-than-male lung cancer incidence during 1983–1992 among 25–44-year-olds in Tasmania, the smallest state of Australia, may yet prove to be a portent of future events.

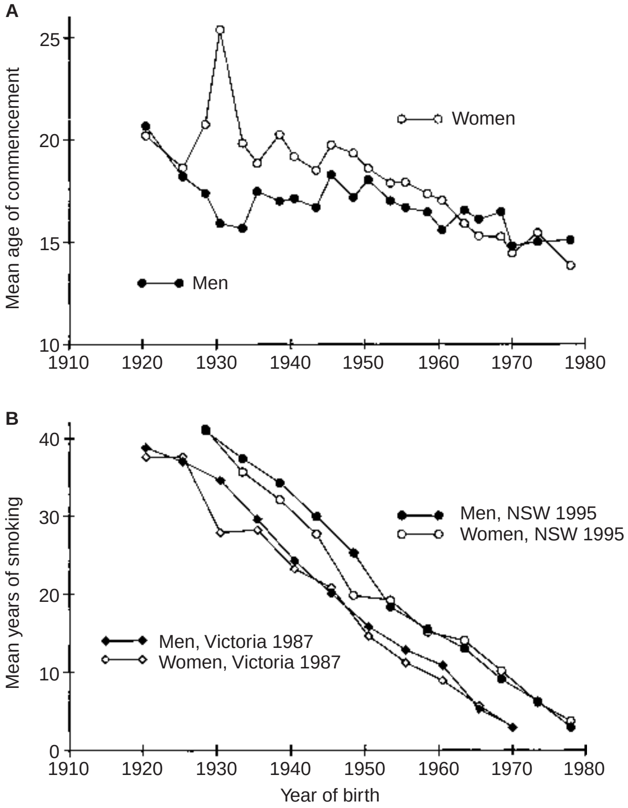

**Figure 5** Smoking by year of birth, Victoria 1987 and New South Wales 1995: mean age of commencement of smoking (**A**), and mean years of smoking (**B**)

The birth cohort analysis of Australian lung cancer mortality showed that the peak in female rates occurred for women born in the 1940s. The declining mortality of successive post-1940s birth cohorts of women is at odds with increasing proportions of ever-smokers, and the increasingly younger ages at which they commenced smoking. The explanation is not early cessation of smoking, which substantially reduces lung cancer risk (Peto et al, 2000). The proportions of female current smokers in subsequent cohorts have not declined, and their years of smoking to a common age have increased. Years of smoking duration was the principal determinant of risk in the study of mainly male British doctors (Doll and Peto, 1978), but the declining lung cancer mortality rates of the post-1940s birth cohorts of Australian women occurred despite longer average duration by increasing numbers of female smokers.

One possibility that could not be investigated with these data was that the trend to lower mortality is due to reductions in the average number of cigarettes smoked daily. If that is the explanation, however, it defies national survey results for women of all ages that daily quantities have changed little between 1980 (Hill and Gray, 1982) and 1995 (Hill et al, 1998). It also runs counter to Australian observations that female smokers born after the 1940s smoked more cigarettes per day, and smoked each cigarette more intensely by inhaling more deeply and discarding less with the butt, than female smokers born in the 1940s.

Another possible explanation is that reduced lung cancer mortality is the outcome of changes in cigarette design. Two major changes were the addition of mainly cellulose acetate filters (‘filter-tips’) in place of the equivalent volume of tobacco for filtration, and reductions in yields of carcinogenic particulate matter (‘tar’) per cigarette. By comparing the timing of those changes with the cohort trends, some tentative inferences are possible.

In Australia, filter-tip cigarettes became popular in the late 1950s. Their market share increased from 1–2% of sales in 1955 to 60% in 1960 and to 75% in 1970 (Walker, 1984). The early filter-tips were high in tar yield, however. The market leader (Rothmans King Size Filter), with 35% of sales in 1963, had a tar yield of 46.0 mg when tested in 1961–1962 (ACA, 1962) compared with 45.8 mg for its equivalent plain version (Rothmans King Size Plain). The Rothmans filter was at least partially effective in filtration, nevertheless, because it reduced machine-measured tar yield by 7% (ACA, 1962). The excess tar yield of the filter-tip was greater for the only two other brands with filter and non-filter versions (Capstan, Craven A), suggesting that different tobacco was used in the early filter-tip cigarettes (ACA, 1962). A number of lower-tar brands were subsequently introduced from 1965, and yields measured with a standard method declined markedly in periodic testing after 1968. Tip perforation (‘ventilation’), which reduces the machine-measured tar yield of cigarettes but less so the tar intake (Evans et al, 1994) of smokers who accidentally or deliberately (Kozlowski et al, 1982) block the holes, was not introduced in Australia until 1975 (Evans et al, 1994).

Australian smokers born in the 1940s commenced smoking when filter-tip cigarettes were first popular but high in tar yield. Most of the women among them smoked filter-tip cigarettes exclusively, but their lung cancer mortality rates were nevertheless markedly higher than the rates of previous birth cohorts of women.

Mortality rates declined first for Australian women born in the 1950s who, if they smoked, did so when Australian-made cigarettes had been reduced in tar yield. Reductions in cigarette tar yields had occurred a decade earlier in the USA and UK, judging from the comparisons of tar yields of US and Australian brands (ACA, 1961) and from trends in average tar yields in the USA and UK (Hoffmann et al, 1994). The lung cancer rates of 20–44-year-old women in the USA and UK commenced to decline in the 1970s (Doll, 1991; Gilliland and Samet, 1994), rather than in the 1980s as in Australia. Reduced risk for smoking the lower tar cigarettes available in the 1950s and 1960s was found in two prospective studies of individuals (Hammond et al, 1976; Stellman and Garfinkel, 1989).

The data analysed in this study are representative of the Australian population. The lung cancer mortality data resulted from complete Australia-wide surveillance, and the smoking prevalence estimates were derived from eight national smoking surveys conducted with a standard method. The analyses of age of commencement and years of smoking were based on data collected by personal interview of 1400 smokers in two states that comprise nearly 60% of the population of Australia.

Nevertheless, there are several limitations of this study. Firstly, the aggregate nature of these data cautions against drawing inferences about reduced risk of lung cancer for individual smokers. Secondly, there were relatively few cases in the post-1940s birth cohorts. The reduction in lung cancer mortality rates for women born in the late 1960s relative to women born in the late 1940s, of around 70% (Figure 3, Panel D), would be good news if true, but the 1960s estimate was based on just two deaths. Thirdly, the post-1940s birth cohorts were observed at younger ages than were the 1940s birth cohorts. To take account of this, the estimated lung cancer mortality rates of birth cohorts were adjusted for age on the assumption and statistical evidence that the effects of age were reasonably constant over birth cohorts. This would not be valid if lung cancer deaths in post-1940s birth cohorts have been postponed to later ages. Fourthly, it was not possible to fully adjust the birth cohort estimates for both age and period effects because of the exact relationship (period = age − cohort) between them (Clayton and Schifflers, 1987). We stratified to control for period, combining age-adjusted estimates of birth cohort effects for two intervals in which period effects were relatively minor. There may have been residual confounding nonetheless.

In conclusion, increased smoking has not resulted in higher lung cancer mortality for Australian women born in the 1950s and 1960s. Their age-adjusted lung cancer mortality rates are lower than those for women born in the 1940s, despite higher proportions of smokers, younger age of commencement and longer duration of smoking in the more recent cohorts. Reductions in tar yields of Australian-made cigarettes, which would have affected primarily those born after the 1940s, may be responsible.

### ACKNOWLEDGEMENTS

The first author was supported in this work by a Public Health Research and Development Committee Research Scholarship awarded by the National Health and Medical Research Council of Australia. The Cancer Council of Tasmania and the WP Holman Clinic at Launceston provided project funding. The authors gratefully acknowledge the assistance provided by Victoria White, Behavioural Scientist at the Anti-Cancer Council of Victoria.

### REFERENCES

Australian Consumers' Association (1961) Australian-made cigarettes: a further report. *Choice* **2**: 68–72

Australian Consumers' Association (1962) ACA's cigarette tests – filter tips: filter – fact or fancy. *Choice* **3**: 145–148

Clayton D and Schifflers E (1987) Models for temporal variation in cancer rates I: Age-period and age-cohort models. *Stats Med* **6**: 449–467

Doll R (1989) Progress against cancer: are we winning the war? *Acta Oncol* **28**: 611–621

Doll R (1991) Progress against cancer: an epidemiologic assessment: the 1991 John C Cassel Memorial Lecture. *Am J Epidemiol* **134**: 675–688

Doll R and Peto R (1978) Cigarette smoking and bronchial carcinoma: dose and time relationships among regular smokers and lifelong non-smokers. *J Epidemiol Comm Health* **32**: 303–133

Dwyer T, Blizzard L, Shugg D, Hill D and Ansari MZ (1994) Higher lung cancer rates in young women than young men: Tasmania, 1983 to 1992. *Cancer Causes Control* **5**: 351–358

Evans GS, Johnson G and Frizzell M (1994) *A study of the smoke yield of vented filter cigarettes*. Australian Government Analytical Laboratories: Melbourne

Gilliland FD and Samet JM (1994) Lung cancer. In: *Cancer surveys: trends in cancer incidence and mortality*. Doll R, Fraumeni JF, Muir CS (eds). Cold Spring Harbor Laboratory Press: New York

Hammond EC, Garfinkel L, Seidman H and Lew EA (1976) 'Tar' and nicotine content of cigarette smoke in relation to death rates. *Environ Res* **12**: 26–74

Hegmann KT, Fraser AM, Keaney RP, Moser SE, Nilasena DS, Sedlars M, Nigham-Gren L and Lyon JL (1993) The effect of age at smoking initiation on lung cancer risk. *Epidemiology* **4**: 444–448

Hill DJ and Gray NJ (1982) Patterns of tobacco smoking in Australia. *Med J Aust* **1**: 23–25

Hill DJ, White VM and Scollo MM (1998) Smoking behaviours of Australian adults in 1995: trends and concerns. *Med J Aust* **168**: 209–213

Hoffmann D, Brunnemann KD, Prokopczyk B and Djordjevic MV (1994) Tobacco-specific N-nitrosamines and Areca-derived N-nitrosamines: chemistry, biochemistry, carcinogenicity, and relevance to humans. *J Toxicol Environ Health* **41**: 1–52

Kozlowski LT, Rickert WS, Pope MA, Robinson JC and Frecker RC (1982) Estimating the yield to smokers of tar, nicotine, and carbon monoxide from the 'lowest yield' ventilated filter-cigarettes. *Br J Addic* **77**: 159–165

Peto R, Darby S, Deo H, Silcocks P, Whitley E and Doll R (2000) Smoking, smoking cessation, and lung cancer risk in the UK since 1950: combination of national statistics with two case-control studies. *BMJ* **321**: 323–329

Stellman SD and Garfinkel L (1989) Lung cancer risk is proportional to cigarette tar yield: evidence from a prospective study. *Prev Med* **18**: 518–525

Walker R (1984) *Under Fire: a history of tobacco smoking in Australia*. Melbourne University Press: Melbourne

Wingo PA, Ries LAG, Giovino GA, Miller DS, Rosenberg HM, Shopland DR, Thun MJ and Edwards BK (1999) Annual report to the nation on the status of cancer, 1973–1996, with a special section on lung cancer and tobacco smoking. *J Natl Cancer Inst* **91**: 675–690

 © 2001 Cancer Research Campaign
